# Elucidating the tolerance mechanisms of faba bean (*Vicia faba* L.) to bean broomrape (*Orobanche crenata Forssk.*) infested soil

**DOI:** 10.1038/s41598-026-59757-4

**Published:** 2026-07-04

**Authors:** Mostafa Abdelkader, Ahmed Elkordy, Khaled Abdelaal, Alaa A. Said, Ahmed R. M. Ridwan

**Affiliations:** 1 Horticulture Department, Faculty of Agriculture, Sohag University, Sohag, 82524 Egypt; 2 Department of Botany and Microbiology, Faculty of Science, Sohag University, Sohag, 82524 Egypt; 3 Department of Agricultural Botany, Faculty of Agriculture, Kafrelsheikh University, Kafrelsheikh, 33516 Egypt; 4 Agronomy Department, Faculty of Agriculture, Sohag University, Sohag, 82524 Egypt

**Keywords:** Legume, *Vicia faba*, *Orobanche crenata*, Root anatomy, Antioxidant defense, Phenolic compounds, Biotechnology, Genetics, Plant sciences

## Abstract

Bean broomrape is one of the most destructive parasitic weeds limiting faba bean production in many Mediterranean and Middle Eastern countries. This study aims to investigate the susceptibility of Faba bean genotypes to Bean Broomrape infestation. A field experiment was conducted to evaluate nine Faba bean cultivars with diverse genetic backgrounds. The tested cultivars were Sakha 1, Misr 3, Mariout 2, Giza 429, Giza 843, Giza 716, Nubaria 1, Nubaria 3, and Wadi 1. Giza 429 had the highest plant (73 cm), and Nubaria 1 had the greatest root fresh weight (39.3 g). Giza 429 produced the highest seed yield (3.2 t/ha) and the lowest Bean Broomrape dry weight (40.3 g/m^2^). In contrast, Nubaria 1 was the most susceptible, with severe yield reduction (0.87 t/ha) and high infestation (110.4 g/m^2^). Physio-biochemical analyses revealed that resistant cultivars (Giza 429, Misr 3) had increased antioxidant activity and phenolic content, while showing reduced proline. Tolerant cultivars had larger vascular cylinder diameters, wider xylem vessels, and thicker phloem tissue, which enhances nutrient transport and structural resistance to Bean Broomrape. Susceptible cultivars displayed reduced vascular development and smaller xylem vessel areas. Giza 429 is a promising genetic resource for breeding programs aimed at enhancing tolerance to broomrape. The findings highlight the potential contributions of biochemical defense responses and root anatomical adaptations to tolerance mechanisms in faba bean under O. crenata infestation.

## Introduction

Legume crops play a crucial role in sustainable agricultural systems because of their ability to establish symbiotic associations with nitrogen-fixing bacteria, thereby enhancing soil fertility and reducing dependence on synthetic nitrogen fertilizers. Legumes are considered key components of low-input and environmentally friendly farming systems, contributing to soil health, crop productivity, and agricultural sustainability^1^. The Mediterranean and East African countries account for about 32% of its global production^2^. Faba bean (*V*. *faba*) is the fourth most important cool-season food legume and grows in more than 60 countries. It has high nutrient content, with plenty of protein, energy, dietary fiber, vitamins, and minerals such as Fe, Cu, Zn, and Mn^3^. It is considered the primary source of amino acids. In addition, its nutritional profile includes antioxidants, phenols, and the amino acid Alanine^4^, as well as various essential amino acids, such as arginine, lysine, and leucine, which are necessary for human diets^4^.

Despite its economic and nutritional importance, faba bean production has declined considerably in several countries, including Egypt. According to FAO statistics, the cultivated area decreased from 77,149 ha, producing 233,523 tons in 2010, to only 23,951 ha, producing 100,316 tons in 2023^5^. Yield reduction is often the main factor limiting Faba bean cultivation in the Mediterranean region^6,7^, which requires improving agronomic practices and developing specific breeding programs adapted to these conditions^8^. Faba bean can be intensively damaged by pests and diseases ^9^, leading to production failure in susceptible genotypes. Weed Bean Broomrape (*O. crenata*) is a primary constraint specific to the Mediterranean region and the Middle East^10^. The penalty on Faba bean yield due to Bean Broomrape infestation depends on the infection level, genotype resistance, and environmental factors^11^. Bean Broomrape is a root-obligate holoparasitic annual plant that infests the secondary roots of susceptible host species. It is also widely distributed across the Mediterranean region^12^. Bean Broomrape belongs to the family Orobanchaceae and infects many dicot plants^13^, causing the most widespread damage to cool-season food legumes in northern Africa, southern Europe, and western Asia^14^, and also occurs sporadically on vegetable crops such as carrot, eggplant, and tomato^15^.

Lacking chlorophyll, *O. crenata* is incapable of independent photosynthesis and growth and therefore relies entirely on its host plants for water, nutrients, and essential metabolites^16^. This parasitic relationship leads to significant yield reductions in host crops and, under severe infestation, can result in complete crop failure. *O. crenata* poses a significant threat to Faba bean production due to the long-term persistence of its seeds in the soil, which remain dormant until their germination is stimulated by a suitable host^17^. Infestation occurs underground, often causing substantial damage to the host plant before Bean Broomrape shoots emerge above ground. The extent of yield loss varies depending on the host’s genotype^11^. Faba bean cultivars exhibit considerable variation in their tolerance to *O. crenata* infection. Tolerant cultivars tend to provide greater resistance to Bean Broomrape parasitism. In addition, these cultivars differ significantly in their morphological and anatomical traits, yield potential, and yield components, including the number of pods per plant and seed yield per unit area^18^. Significant research efforts have been made to develop resistant or tolerant cultivars of legumes and industrial crops against broomrape^17^. The researcher observed that Faba bean genotypes with greater root and shoot biomass tended to exhibit higher infestation density of *Orobanche* tubercles, through a mechanism involving reduced strigolactone exudation from plant roots^19,20^.

Resistance to Bean Broomrape in Faba bean and other legumes involves multiple defense mechanisms. Lignification occurs after initial parasite penetration is halted in the cortex, thereby preventing further intrusion into the vascular system and haustorium formation^21^. The resistant cultivars consistently exhibited lower *Orobanche* spike counts than susceptible ones^22^. A limited number of studies have explored genetic diversity as a potential source of resistance to parasitic weeds^23^. A deeper understanding of these mechanisms is essential for developing effective breeding strategies and improving broomrape management in faba bean production systems^17^.

Therefore, the present study aimed to evaluate the response of nine faba bean cultivars to natural infestation by O. crenata by assessing growth attributes, yield performance, biochemical characteristics, and root anatomical traits. Furthermore, the study sought to identify key traits associated with broomrape tolerance and to determine promising cultivars that could serve as valuable genetic resources for future breeding programs aimed at enhancing faba bean productivity under broomrape-infested conditions.

## Materials and methods

### Experimental design

A field experiment was conducted at the Agricultural Experimental Station of the Faculty of Agriculture, Sohag University, Egypt, during the 2024/2025 growing season. The experiment included nine Faba bean cultivars as treatments. The experiment was arranged in a randomized complete block design (RCBD) with three replications and a plot area of 10.5 m^2^. The Faba bean genotypes cultivated in this study originate from diverse genetic backgrounds (Table 1). Sakha 1 was developed from a cross between lines 716/724/88 and 620/283/85. Misr 3 resulted from a cross between line 667 and a hybrid of Cairo 241 and Giza 461. Mariout 2 was developed by hybridizing two Egyptian Faba bean cultivars at the Mariout Agricultural Research Station, affiliated with the Desert Research Center in Egypt. Giza 429 was obtained by individual plant selection from Giza 402. Giza 843 was bred from a cross between lines 461/854/83 and 561/2076/85, while Giza 716 originated from a cross between lines 461/842/83 and 503/453/83. Nubaria 1 and Nubaria 3 were selected through local selection programs in Rena Blanka and Ahnasiaz, respectively. Lastly, Wadi 1 was developed from a cross between Rena Blanka and Triple White. Seeds of all faba bean genotypes were provided by the Food Legumes Research Department, Agricultural Research Center (ARC), Giza, Egypt.

**Table 1:** List of cultivated Faba bean genotypes and their source and pedigree.

Cultivars	Source	Pedigree
Sakha 1	ARC, Giza	716/724/88 × 620/283/85
Misr 3	ARC, Giza	667x (Cairo 241 × Giza 461)
Mariout 2	ARC, Giza	Developed through hybridization between two Egyptian faba bean cultivars at the Mariout Agricultural Research Station, part of the Desert Research Center, Egypt
Giza 429	ARC, Giza	Individual plant selection from Giza 402
Giza 843	ARC, Giza	461/854/83 × 561/2076/85
Giza 716	ARC, Giza	461/842/83 × 503/453/83
Nubaria 1	ARC, Giza	Selection in Rena Blanka
Nubaria 3	ARC, Giza	Selection in Ahnasiaz
Wadi 1	ARC, Giza	Rena Blanka x Triple white

### Soil Characteristics

Composite soil samples (0–30 cm depth) were collected from the experimental field before sowing and analyzed according to standard procedures. The soil had a sandy loam texture, consisting of 65% sand, 16% silt, and 19% clay, with 7.31% gravel content. Soil organic matter content was 1.31%, total nitrogen was 0.73%, available phosphorus (P₂O₅) was 19.5 ppm, and available potassium (K₂O) was 141.72 ppm. The soil was slightly alkaline (pH 7.82) with an EC of 1.12 dS m⁻^1^.

## Measurement

### Growth and Yield Traits

Growth parameters were evaluated at 60 days after sowing using randomly selected guarded plants from each experimental plot, as described by ^24^. The measured traits included plant height (cm), number of branches per plant, root fresh weight (g plant⁻^1^), main root length (cm), main root diameter (cm), and number of secondary roots. In addition, the number of days to 50% flowering and physiological maturity was recorded for each cultivar. At harvest, representative plant samples were randomly collected from each plot to determine yield and yield-related traits. The evaluated parameters included plant height (cm), number of branches per plant, number of pods per plant, number of seeds per pod, 100-seed weight (g), seed yield per plant (g), biological yield (t ha⁻^1^), seed yield (t ha⁻^1^), straw yield (t ha⁻^1^), and harvest index (%).

The degree of broomrape infestation was assessed by recording the number of Orobanche crenata spikes per square meter (spikes m⁻^2^) and the dry weight of broomrape spikes (g m⁻^2^).

### Biochemical Traits

Chlorophyll a, b, and carotenoids were extracted with 80% acetone, then quantified by colorimetric analysis using a Shimadzu UV-240 UV/VIS spectrophotometer (Japan) ^24^. Proline was extracted from fresh Plant leaves with 3% sulfosalicylic acid. Acid ninhydrin and glacial acetic acid were added to each sample. The mixture was incubated for 1 h in a boiling water bath, then cooled in an ice bath. Finally, toluene was added to the mixture, and the absorbance was measured at 520 nm using a spectrophotometer ^25^. The phenolic content in leaves and fruits was measured using the Folin-Ciocalteu reagent method. This involved spectrophotometric analysis at 765 nm ^26^. Flavonoid content in plant leaves and fruits was measured using aluminum chloride and potassium acetate, with spectrophotometric analysis conducted at 415 nm ^27,28^.

Total antioxidant activity was determined in plant leaves and fruits by the 1,1-diphenyl-2-picrylhydrazyl (DPPH) method. A stock solution was prepared by dissolving 24 mg of DPPH in 100 mL of methanol and stored at 20 °C. The working solution was diluted to 10 ml with 45 ml of methanol to achieve an absorbance of 1.1 ± 0.02 at 515 nm. Extracts (750 μL) were mixed with 1,500 μL of DPPH solution and incubated in the dark for 5 min, after which absorbance was measured at 515 nm. The standard curve was linear between 25 and 800 μmol Trolox ^29^. The assay mixture was prepared with pyrogallol and H_2_O_2_ according to the Sigma Manual. The change in absorbance was continuously monitored at 420 nm. One unit of enzyme activity was defined as a 0.001/min change in absorbance ^30^.

### Root Anatomy

Fully mature roots were selected. Fresh roots were preserved in formalin, glacial acetic acid (FAA), and 70% Ethyl alcohol. The samples were dehydrated using increasing concentrations of ethyl alcohol. Using a Leitz 1512 microtome, the top third of the roots has been sectioned at 20–30 μm. After staining with safranin for 30 min, the tissue was rinsed with 50%, 70%, and 95% EtOH, respectively, for 3–5 min each. Light green (1%) stains plant tissues intensely in just 30 s ^31^. The tissue was rinsed with 95%, 100% EtOH, and 99% xylene for 3–5 min each. Canada Balsam, a mounting medium, stabilized the tissue before a slide cover was placed over it. The prepared slides were dried in a hot air oven at 55°C for 48 h to ensure proper fixation. Five to ten slides were prepared for each cultivar. Sections were examined, and Photomicrographs were taken with a compound microscope (Olympus SZX7) at the Herbarium, Botany and Microbiology Dep, Sohag University. All quantitative measurements of root cross-sections were collected using ImageJ v1.45. Mean quantitative measurements were conducted based on at least five readings from each slide. Terminology was used to describe root anatomy ^32^.

### Statistical analysis

The experiment was arranged in a randomized complete block design (RCBD) with three replications. Data were analyzed using the General Linear Model (GLM) procedure in SAS software (Version 9.1; SAS Institute Inc., Cary, NC, USA) with an analysis of variance (ANOVA) ^33^. Treatment means were compared using Duncan’s Multiple Range Test (DMRT) at a significance level of P ≤ 0.05. Principal component analysis (PCA) and correlation were performed in R to investigate relationships among the studied traits and evaluate the performance of the faba bean cultivars.

### Results

 The ANOVA revealed highly significant differences (P ≤ 0.05 to P ≤ 0.0001) among faba bean genotypes for most of the traits studied (Table 2). Strong genetic variability and differential responses under naturally infested conditions were noticed in this experiment. Significant variation was observed among the agronomic traits studied, confirming that genotype plays a major role in determining productivity under broomrape stress. Infestation parameters, biochemical markers, and antioxidant enzyme activities also showed highly significant differences. This highlights diverse defense responses among the cultivated genotypes. In contrast, no significant differences were observed for plant height, number of branches per Plant, and chlorophyll b content under the experimental conditions. The low-to-moderate coefficients of variation (CV%) across most traits indicate acceptable experimental precision. At the same time, the significant F-values confirm the reliability of the observed treatment effects and the robustness of the experimental design in discriminating between tolerant and susceptible genotypes.

**Table 2:** Summary of ANOVA for the studied traits of the cultivated faba bean genotypes under broomrape natural infection.

Trait	SS Treatments	MS Treatments	SS Rep	SS Error	MS Error	F-value	P-value	Sign	CV%	SE ±	LSD_0.05_
Plant height (cm)	925.99	115.75	70.087	869.55	54.347	2.130	0.0943	n.s	12.01%	4.25	-
No. branches/Plant	14.667	1.833	0.222	11.778	0.736	2.491	0.0573	n.s	24.13%	0.49	-
Days to flowering	267.11	33.389	0.519	7.259	0.454	73.592	< 0.0001	**	1.25%	0.38	1.16
Days to maturity	109.27	13.658	0.601	6.288	0.393	34.754	< 0.0001	**	0.41%	0.36	1.08
Fresh weight of the root	894.44	111.81	31.247	322.28	20.142	5.551	0.0018	**	16.39%	2.59	7.76
Main root length	106.25	13.282	41.687	80.347	5.022	2.645	0.0465	*	15.84%	1.29	3.87
Main root diameter	2.021	0.253	0.005	0.788	0.049	5.128	0.0027	**	14.00%	0.12	0.38
No. secondary roots	1275.41	159.43	20.519	737.48	46.093	3.459	0.0166	*	23.29%	3.91	11.75
100 seeds Weight	497.94	62.243	164.77	359.93	22.496	2.767	0.0396	*	6.14%	2.73	8.20
Seed yield /plant (g)	621.74	77.717	9.241	103.03	6.439	12.069	< 0.0001	**	20.13%	1.46	4.39
Biological yield	75.886	9.486	3.814	34.650	2.166	4.380	0.0058	**	20.09%	0.84	2.54
Seed yield (ton/ha)	12.996	1.625	0.149	2.119	0.132	12.266	< 0.0001	**	15.64%	0.21	0.62
Straw yield (ton/ha)	39.739	4.967	2.624	20.888	1.306	3.805	0.0110	*	22.87%	0.65	1.97
Harvest index (%)	1470.96	183.87	3.649	104.36	6.523	28.189	< 0.0001	**	7.89%	1.47	4.42
No of broomrapes	8411.63	1051.45	168.07	488.59	30.537	34.432	< 0.0001	**	13.94%	3.19	9.56
Broomrape dry weight	10,473.73	1309.22	5.887	306.15	19.134	68.423	< 0.0001	**	6.69%	2.52	7.57
Total Phenolic	6331.93	791.49	0.006	287.86	17.991	43.994	< 0.0001	**	6.17%	2.44	7.34
Total Flavonoids	0.491	0.061	0.019	0.022	0.001	44.002	< 0.0001	**	1.68%	0.02	0.06
DPPH	61.047	7.631	11.215	38.994	2.437	3.131	0.0248	*	2.29%	0.90	2.70
Proline	1587.28	198.41	48.564	221.45	13.841	14.335	< 0.0001	**	27.20%	2.14	6.43
Polyphenol oxidase	10,515.87	1314.48	646.56	2040.27	127.52	10.308	0.0001	**	23.15%	6.51	19.54
Peroxidase	1163.91	145.49	33.511	175.33	10.958	13.277	< 0.0001	**	8.40%	1.91	5.72
Chlorophyll a	0.175	0.022	0.037	0.079	0.005	4.434	0.0055	**	0.75%	0.04	0.12
Chlorophyll b	25.200	3.150	17.013	22.667	1.417	2.223	0.0827	n.s	23.41%	0.68	-

** Highly significant (P < 0.01); * Significant (P < 0.05); n.s. Not significant; RCBD: df (Treatments) = 8, df (Rep) = 2, df (Error) = 16.

### Growth and Yield Traits

The statistical analysis revealed significant differences among the evaluated faba bean cultivars in key growth and phenological traits (Table 3), indicating substantial genetic variability in their developmental performance under field conditions. At 60 days after sowing, plant height varied markedly among cultivars. Giza 429 recorded the greatest plant height (73.0 cm), indicating superior early vegetative growth, whereas Mariout 2 exhibited the shortest plants (52.8 cm), reflecting comparatively weaker early vigor under the prevailing conditions. Such variation in plant stature suggests differential growth potential and resource utilization efficiency among the tested genotypes. Phenological development also showed clear cultivar-dependent variation. Nubaria 1 required the longest duration to reach 50% flowering (58.88 days), indicating a relatively delayed reproductive transition, while Wadi 1 was the earliest to flower, reaching 50% flowering at 48.33 days. This wide range in flowering time reflects distinct developmental pacing and may influence adaptation to environmental constraints, particularly in regions with terminal stress conditions. A similar pattern was observed for maturity. Wadi 1 reached physiological maturity earlier than all other cultivars (149.67 days), whereas Giza 716 exhibited the longest growth duration, reaching maturity at 155.44 days. The differences in maturity duration further confirm the existence of contrasting growth strategies among the cultivars, ranging from early-maturing types to relatively late-maturing genotypes with extended vegetative and reproductive phases.

The data in Table 3 reveal considerable differences in root morphological traits among the studied Faba bean cultivars. The evaluated faba bean genotypes exhibited significant variation in root morphological characteristics under broomrape-infested conditions. This indicates genotypic differences in root system architecture and potential adaptation mechanisms. Nubaria 1 and Wadi 1 recorded the highest fresh root weight, main root diameter, and number of secondary roots. In contrast, tolerant cultivars such as Giza 429 and Giza 843 showed lower root biomass and fewer secondary roots than the highly susceptible genotypes, which may reduce opportunities for Orobanche crenata attachment and establishment. Regarding root length, Mariout 2, Nubaria 3, and Wadi 1 exhibited significantly longer main roots, whereas Misr 3 and Giza 716 had relatively shorter roots. However, tolerance to broomrape infestation was not directly associated with a single root trait, as tolerant genotypes combined moderate root development with potentially more effective structural and physiological defense mechanisms.

**Table 3:** Growth and yield traits of Faba bean cultivars under naturally infested soil conditions by Bean Broomrape.

Parameter	Sakha 1	Misr 3	Mariout 2	Giza 429	Giza 843	Giza 716	Nubaria 1	Nubaria 3	Wadi 1
Plant height (cm)	53.16 ± 3.74^b^	59.00 ± 3.78^ab^	52.80 ± 2.19^b^	73.00 ± 2.51^a^	63.66 ± 7.51^ab^	61.16 ± 1.36^ab^	61.50 ± 2.75^ab^	66.00 ± 6.11^ab^	61.90 ± 3.72^ab^
Number of branches/Plant	2.66 ± 0.33^bc^	2.33 ± 0.33^c^	4.00 ± 0.57^abc^	3.66 ± 0.33^abc^	3.00 ± 0.57^abc^	3.33 ± 0.33^abc^	4.66 ± 0.66^a^	4.00 ± 0.57^abc^	4.33 ± 0.33^ab^
Days to 50% flowering	55.77 ± 0.29^b^	55.22 ± 0.22^bc^	52.00 ± 0.19^d^	50.44 ± 0.22^e^	54.22 ± 0.11^c^	56.11 ± 0.55^b^	58.88 ± 0.58^a^	51.00 ± 0.50^de^	48.33 ± 0.38f.
Number of day to Maturity	154.78 ± 0.11^a^	151.00 ± 0.19^ cd^	151.11 ± 0.22^ cd^	150.22 ± 0.11^de^	153.56 ± 0.22^b^	155.44 ± 0.22^a^	150.00 ± 0.38^de^	151.78 ± 0.80^c^	149.67 ± 0.38^e^
Fresh weight of root (g)	21.40 ± 2.05^b^	25.30 ± 1.30^b^	22.93 ± 0.53^b^	25.26 ± 1.18^b^	24.23 ± 2.14^b^	25.76 ± 1.41^b^	39.33 ± 5.29^a^	26.03 ± 1.78^b^	36.13 ± 3.66^a^
Main root length (cm)	13.50 ± 0.28^ab^	11.16 ± 0.60^b^	16.66 ± 1.87^a^	13.76 ± 1.36^ab^	15.00 ± 2.08^ab^	11.66 ± 1.33^b^	12.70 ± 0.88^ab^	16.83 ± 2.61^a^	16.00 ± 0.86^a^
Main root diameter (cm)	1.20 ± 0.05^d^	1.50 ± 0.10^bcd^	1.40 ± 0.10^dc^	1.66 ± 0.14^abc^	1.50 ± 0.25^bcd^	1.86 ± 0.08^ab^	1.93 ± 0.08^a^	1.23 ± 0.06^d^	1.96 ± 0.06^a^
No. secondary roots	20.00 ± 1.15^c^	27.33 ± 2.84^bc^	26.66 ± 2.84^bc^	28.33 ± 2.90^bc^	22.00 ± 1.15^c^	26.33 ± 1.85^bc^	42.33 ± 8.96^a^	30.33 ± 1.76^abc^	39.00 ± 3.46^ab^
Weight of 100 seeds (g)	80.45 ± 3.75^ab^	69.70 ± 3.08^c^	75.14 ± 2.22^abc^	76.27 ± 2.78^abc^	79.71 ± 5.47^ab^	72.31 ± 1.20^bc^	81.62 ± 1.08^a^	75.53 ± 2.01^abc^	83.65 ± 3.83^a^
Seed yield per plant (g)	15.21 ± 0.71^b^	9.34 ± 1.35^cd^	12.95 ± 1.58^bc^	20.88 ± 2.30^a^	17.41 ± 1.08^ab^	9.66 ± 1.69^cd^	4.71 ± 0.35^e^	15.15 ± 1.43^b^	8.06 ± 1.53^de^
Biological yield (ton/ha)	7.73 ± 0.46^ab^	5.37 ± 0.68^b^	6.54 ± 0.59^b^	10.40 ± 1.39^a^	8.20 ± 0.82^ab^	6.34 ± 0.93^b^	5.59 ± 0.30^b^	9.59 ± 0.91^a^	6.15 ± 1.00^b^
Straw yield (ton/ha)	4.94 ± 0.40^bc^	3.30 ± 0.49^c^	4.08 ± 0.44^c^	7.22 ± 1.09^a^	5.24 ± 0.74^abc^	4.03 ± 0.64^c^	4.72 ± 0.29^bc^	6.83 ± 0.78^ab^	4.60 ± 0.70^c^
Harvest Index (%)	37.17 ± 0.64^a^	39.61 ± 1.36^a^	38.11 ± 1.16^a^	31.40 ± 1.34^b^	37.45 ± 1.99^a^	37.55 ± 0.89^a^	16.32 ± 0.94^d^	29.07 ± 1.33^b^	24.63 ± 2.28^c^
No of broomrape (Spikes/m^2^)	21.33 ± 4.10^e^	39.00 ± 3.61^c^	36.33 ± 2.40^cd^	27.67 ± 2.91^de^	23.33 ± 3.53^e^	58.00 ± 5.29^b^	68.33 ± 2.85^a^	20.33 ± 3.18^e^	62.33 ± 2.60^ab^
Dry weight of Broomrape (g/m^2^)	42.48 ± 2.49^e^	71.06 ± 1.36^bc^	75.86 ± 1.73^b^	40.29 ± 3.46^e^	52.75 ± 2.22^d^	64.69 ± 2.61^c^	110.38 ± 1.12^a^	63.20 ± 2.60^c^	67.64 ± 3.04^c^

Data are presented as mean ± standard error (SE). Means within the same row carrying different superscript letters are significantly different at P < 0.05 based on Duncan’s multiple range test (DMRT).

The lower number of secondary roots observed in Giza 429 and Giza 843 may reduce the number of parasite infection sites, thereby contributing to lower broomrape spike density and reduced parasite biomass accumulation. Giza 429 produced the highest seed yield per Plant at 20.88 g, followed by Giza 843 at 17.41 g (Table 3). In contrast, Nubaria 1 had the lowest yield, producing just 4.71 g per plant. Regarding biological yield, Giza 429 again led with 10.40 t/ha, while Misr 3 had the lowest at 5.37 t/ha. Under natural Bean Broomrape infestation, Giza 429 maintained its position as the top-performing cultivar, achieving a seed yield of 3.18 t/ha, closely followed by Giza 843 at 2.96 t/ha.

Nubaria 1 was the most affected by bean broomrape, managing only 0.87 t/ha under infestation, highlighting its high susceptibility. Regarding straw yield, Giza 429 also performed best, followed by Nubaria 3. Misr 3 and Giza 716 recorded the lowest straw yields. Interestingly, while Misr 3 had the highest harvest index. Significant differences among Faba bean cultivars in their responses to natural Bean Broomrape infestation, particularly in spike number and dry weight (Table 3). Nubaria 1 was the most heavily affected, with the highest infestation (68.33 spikes per square meter) and a Bean Broomrape dry weight of 110.38 g/m^2^, highlighting its high susceptibility. In contrast, Nubaria 3 showed the lowest spike count, with just 20.33 spikes per square meter, indicating better resistance. Meanwhile, Giza 429 recorded the lowest Bean Broomrape dry weight at 40.29 g/m^2^. These results indicate that Giza 429 likely has built-in tolerance mechanisms that help it withstand Bean Broomrape infection, possibly due to specific traits that limit the parasite’s adverse effects. As a result, Giza 429 is a strong candidate for cultivation in Bean Broomrape-infested areas.

### Physio-Biochemical Traits

Total phenolic content (TPC) varied widely, ranging from 43.2 mg/g in Sakha 1 to 94.3 mg/g in Misr 3. The highest phenolic levels were recorded in Misr 3 and Nubaria 1, suggesting that these genotypes activate strong chemical defense pathways, particularly the phenolic compound pathway, which is often linked to resistance to parasitic weeds such as *O. crenata* ^34^. In contrast, Sakha 1 had the lowest phenolic content, indicating a weaker biochemical defense and greater susceptibility to Bean Broomrape infestation (Table 4).

**Table 4:** Physio-biochemical studied traits of Faba bean leaves cultivated under broomrape-infested soil.

Parameter	Total Phenolic	Total Flavonoids	DPPH	Proline	Polyphenol oxidase	Peroxidase	Chlorophyll a	Chlorophyll b
Sakha 1	43.17 ± 2.92d	2.28 ± 0.03b	67.87 ± 1.50ab	24.35 ± 1.79ab	34.39 ± 1.01cd	36.03 ± 0.65cd	9.28 ± 0.002c	5.49 ± 0.30ab
Misr 3	94.26 ± 3.64a	2.22 ± 0.03bc	68.64 ± 0.06ab	5.81 ± 0.59d	59.46 ± 15.59b	42.07 ± 0.92bc	9.35 ± 0.017bc	4.91 ± 0.02ab
Mariout 2	59.51 ± 2.63c	2.27 ± 0.01bc	67.97 ± 1.44ab	9.01 ± 0.10d	37.32 ± 1.85cd	35.40 ± 3.37d	9.26 ± 0.043c	6.31 ± 0.92a
Giza 429	72.65 ± 1.44b	2.28 ± 0.03b	70.27 ± 0.44a	7.86 ± 0.28d	39.84 ± 1.78bcd	45.08 ± 0.32ab	9.32 ± 0.067bc	6.05 ± 1.50a
Giza 843	55.98 ± 0.53c	2.01 ± 0.02e	66.82 ± 0.78b	10.87 ± 0.36d	25.35 ± 0.68d	25.95 ± 0.05e	9.31 ± 0.038bc	5.53 ± 0.69ab
Giza 716	68.31 ± 1.69b	2.50 ± 0.03a	66.43 ± 1.33b	12.30 ± 1.09cd	43.86 ± 0.61bcd	40.75 ± 4.36bcd	9.53 ± 0.042a	3.21 ± 0.57b
Nubaria 1	90.25 ± 1.07a	2.21 ± 0.01c	70.27 ± 0.67a	6.43 ± 1.68d	97.40 ± 0.63a	50.02 ± 1.15a	9.44 ± 0.004ab	3.70 ± 0.26b
Nubaria 3	60.82 ± 1.13c	2.08 ± 0.04de	65.76 ± 0.39b	28.39 ± 6.06a	46.82 ± 8.18bcd	36.08 ± 1.32cd	9.36 ± 0.065bc	5.48 ± 0.86ab
Wadi 1	72.82 ± 3.42b	2.12 ± 0.03d	67.10 ± 0.94b	18.03 ± 0.67bc	54.45 ± 11.36bc	43.23 ± 0.17b	9.37 ± 0.073bc	5.06 ± 1.35ab

Data are presented as mean ± standard error (SE). Means within the same column carrying different, superscript letters significantly differ at P < 0.05 based on Duncan’s multiple range test (DMRT).

Giza 716 showed the highest flavonoid level (2.50 mg/g), which may help neutralize harmful reactive oxygen species (ROS) generated under stress. Giza 843, with the lowest TFC (2.01 mg/g), may lack this additional protective mechanism. The DPPH radical scavenging assay, used to assess antioxidant capacity, revealed that Giza 429 and Nubaria 1 had the highest activity levels (70.27%), followed by Misr 3 (68.64%). These values indicate strong antioxidant systems in these genotypes, enabling them to better cope with oxidative stress induced by Bean Broomrape. In contrast, Nubaria 3 and Giza 716 had the lowest antioxidant activity. Nubaria 3 (28.39 µmol/g) and Sakha 1 (24.35 µmol/g) accumulated the highest proline amounts, indicating increased stress under infestation. Conversely, Misr 3, Nubaria 1, and Giza 429 had much lower proline levels. Giza 429 and Misr 3 were the most promising candidates for resistance to bean broomrape.

Polyphenol oxidase (PPO) activity varied widely, ranging from 25.35 U/g FW in Giza 843 to 97.40 U/g FW in Nubaria 1. This elevated PPO activity in Nubaria 1 suggests a strong oxidative defense response, likely contributing to cell wall lignification and limiting parasite intrusion. In contrast, the low PPO activity in Giza 843 may indicate a weaker defense mechanism (Table 4). Peroxidase (POD) activity followed a similar pattern. Nubaria 1 again recorded the highest level (50.02 U/g FW), followed closely by Giza 429 (45.08 U/g FW) and Wadi 1 (43.23 U/g FW). High POD levels are often linked to improved stress tolerance because they break down hydrogen peroxide and reinforce cell walls. Giza 843, on the other hand, showed the lowest POD activity (25.95 U/g FW). As for chlorophyll content, values remained relatively stable across cultivars, ranging from 9.26 to 9.53 mg/g FW, with Giza 716 showing the highest total chlorophyll content. In comparison, chlorophyll b content showed greater differences. Mariout 2 (6.31 mg/g FW) and Giza 429 (6.05 mg/g FW) had the highest levels (Table 4).

### Root Anatomical features

**Fig. 1:**
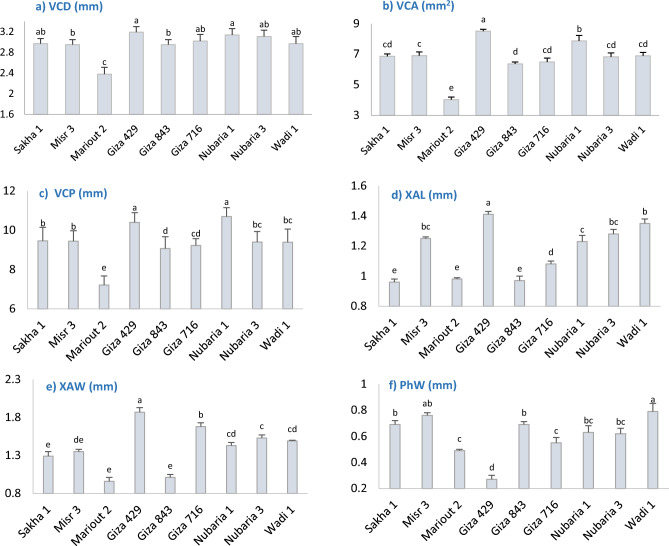
Summary of the studied cultivars’ main root quantitative anatomical measurements. (**a**) VCD = Vascular Cylinder Diameter, (**b**) VCA = Vascular Cylinder Area, (**c**) VCP = Vascular Cylinder Perimeter, (**d**) XAL = Xylem Arm Length, (**e**) XAW = Xylem Arm Width, (**f **) PhW = Phloem width. Data are presented as mean ± standard error (SE). In each chart, Means carry the same letters; they differ non-significantly at P < 0.05 based on Duncan’s multiple range test (DMRT).

Root Root anatomical traits showed marked variation among the studied faba bean cultivars, particularly in vascular cylinder development and related structural components (Fig. 1). The vascular cylinder diameter (VCD) of the main root ranged from 2.38 mm in ‘Mariout 2’ to 3.19 mm in ‘Giza 429’, indicating clear genotypic differences in central vascular development. In lateral roots, VCD varied more widely, ranging from 1.47 mm in ‘Misr 3’ to 3.29 mm in ‘Giza 429’, with the latter exhibiting consistently higher values across both root types. Vascular cylinder area (VCA) of the main root ranged from 4.03 mm^2^ in ‘Mariout 2’ to 8.50 mm^2^ in ‘Giza 429’, while lateral root VCA varied from 1.63 mm^2^ in ‘Misr 3’ to 7.65 mm^2^ in ‘Giza 429’. These results further confirm the superior vascular development of ‘Giza 429’ compared with the other cultivars and suggest substantial genetic variability in root conductive tissue allocation.

Vascular cylinder perimeter (VCP) also differed among cultivars. In the main roots, VCP ranged from 7.21 mm in ‘Mariout 2’ to 10.70 mm in ‘Nubaria 1’, with a mean value of 9.37 mm. In lateral roots, values ranged from 4.58 mm in ‘Misr 3’ to 9.95 mm in ‘Giza 429’, with an overall mean of 5.67 mm. Notably, ‘Giza 429’ exhibited nearly similar perimeters in main (10.41 mm) and lateral roots (9.95 mm), indicating a more balanced vascular architecture compared with the other cultivars. Xylem arm length (XAL) in the main root ranged from 0.96 mm in ‘Sakha 1’ to 1.41 mm in ‘Giza 429’, with a mean of 1.17 mm. In lateral roots, XAL was generally lower, ranging from 0.56 mm in ‘Misr 3’ to 1.55 mm in ‘Giza 429’. Although most cultivars followed the expected pattern of longer xylem arms in the main root, ‘Giza 429’ represented an exception, where the lateral root exhibited slightly greater xylem arm length than the main root.

**Fig. 2:**
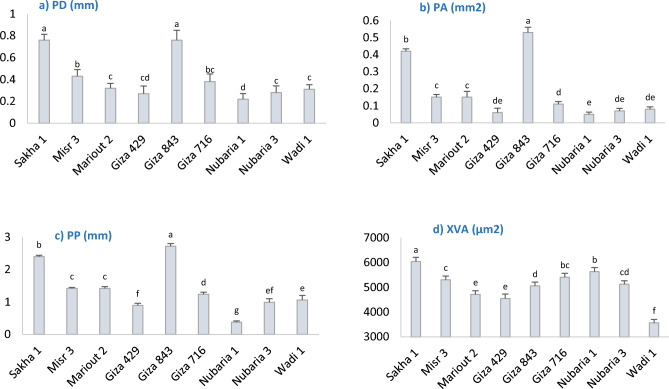
Summary of the studied cultivars’ main root quantitative anatomical measurements. (**a**) PD = Pith Diameter, (**b**) PA = Pith area, (** c**) PP = Pith Perimeter, (** d**) XVA = Xylem Vessels Area. Data are presented as mean ± standard error (SE). Means in each chart carry the same letters that do not significantly differ at P < 0.05 based on Duncan’s multiple range test (DMRT).

Xylem arm width (XAW) showed a similar trend, with values in main roots ranging from 0.96 mm in ‘Mariout 2’ to 1.87 mm in ‘Giza 429’ (mean = 1.40 mm). In lateral roots, XAW ranged from 0.57 mm in ‘Nubaria 1’ to 1.45 mm in ‘Giza 429’, with a mean of 0.89 mm. The consistently higher xylem dimensions observed in ‘Giza 429’ suggest enhanced development of water- and assimilate-conducting tissues, reflecting strong genetic control over vascular differentiation. Phloem width (PhW) also varied significantly among cultivars. In the main roots, it ranged from 0.27 mm in ‘Giza 429’ to 0.79 mm in ‘Wadi 1’, with a mean value of 0.61 mm. In lateral roots, phloem width was narrower, ranging from 0.19 mm in ‘Giza 843’ to 0.56 mm in ‘Wadi 1’, with a mean of 0.38 mm. Across most cultivars, the phloem region was consistently wider in main roots than in lateral roots, indicating a general structural pattern in vascular allocation see Fig. 2.

Pith diameter (PD) in main roots also showed clear variation among cultivars (Fig. 2), ranging from 0.22 mm in ‘Nubaria 1’ to 0.76 mm in both ‘Giza 843’ and ‘Sakha 1’. This variability further reflects differences in central root anatomical organization and contributes to the observed diversity in root structural traits among the evaluated genotypes. Pith area (PA) in the main roots exhibited clear variation among cultivars, ranging from 0.05 mm^2^ in ‘Nubaria 1’ to 0.53 mm^2^ in ‘Giza 843’. Several cultivars, including ‘Nubaria 1’ (0.05 mm^2^) and ‘Giza 843’ (0.06 mm^2^ in some observations), showed relatively small pith areas, indicating reduced development of central parenchymatous tissue within the root stele. In lateral roots, the pith was absent in most cultivars, except ‘Misr 3’, which exhibited a measurable pith area of 0.04 mm^2^. This widespread absence of pith tissue in lateral roots suggests a general reduction or complete suppression of central parenchyma development in these root types under the studied conditions.

**Fig. 3:**
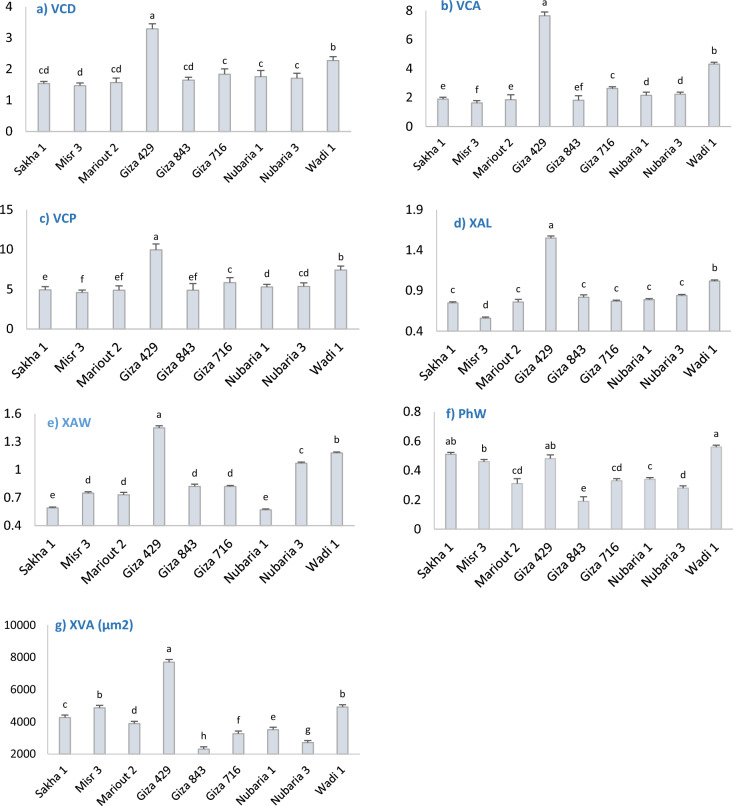
Summary of the studied cultivars’ lateral root quantitative anatomical measurements. (**a**) VCD = Vascular Cylinder Diameter, (**b**) VCA = Vascular Cylinder Area, (**c**) VCP = Vascular Cylinder Perimeter, (**d**) XAL = Xylem Arm Length, (**e**) XAW = Xylem Arm Width, (** f**) PhW = Phloem width, (**g**) XVA = Xylem Vessels Area. Data are presented as mean ± standard error (SE). In each chart, Means carry the same letters; they differ non-significantly at P < 0.05 based on Duncan’s multiple range test (DMRT).

Pith perimeter (PP) in main roots ranged from 0.83 mm in ‘Nubaria 1’ to 2.72 mm in ‘Giza 843’. Relatively high values were also recorded in ‘Giza 716’, ‘Misr 3’, and ‘Sakha 1’, indicating a more expanded central pith region in these cultivars. In contrast, lateral roots showed a complete absence of measurable pith perimeter in all cultivars except ‘Misr 3’, which recorded a small value of 0.75 mm. This further confirms the structural simplification of lateral roots, characterized by reduced or absent central parenchymatous tissues. Xylem vessel area (XVA) also showed pronounced variation between root types and cultivars. In general, the main roots exhibited larger xylem vessel areas compared with the lateral roots, reflecting their primary role in water and nutrient transport. However, exceptions were observed in ‘Giza 429’ and ‘Wadi 1’, where lateral roots displayed larger xylem vessel areas than main roots (Fig. 3), suggesting cultivar-specific adaptations in vascular function and resource transport capacity.

The mean xylem vessel area in main roots ranged from 3570 µm^2^ in the cultivar of Wadi 1to 6038 µm^2^ in the Sakha 1 cultivar, while in lateral roots, values ranged from 2308 µm^2^ in the Giza 843 cultivar to 7699 µm^2^ in the Giza 429 cultivar. The highest main root vessel area variability was observed in Giza 429 Cultivar, displaying the greatest main root vessel enlargement anatomy (Fig. 3). The obtained data reveal significant genotypic variation in lateral root pith characteristics as Misr 3 being the only one exhibiting measurable pith dimensions (PD = 0.26 mm, PA = 0.04 mm^2^, PP = 0.75 mm), while all other genotypes (Sakha 1, Mariout 2, Giza 429, Giza 843, Giza 716, Nubaria 1, Nubaria 3, Wadi 1) show absent pith structures.

### Multivariate analysis

Principal Component Analysis (PCA) was used to identify traits contributing to variation among the studied faba bean genotypes under Orobanche crenata infestation ^35^. The eigenvalue analysis revealed that the first and second principal components (PC1 and PC2) accounted for 69.90% and 23.60% of the total variation, respectively, and together explained 93.5% of the observed variability (Table 5). This high cumulative variance indicates that the first two components capture the relationships among genotypes and the traits under study, making the PCA biplot a reliable representation of the multivariate dataset. Table 5 shows that PC1 had a markedly greater eigenvalue (23.7) than the remaining components, suggesting that it accounts for the greatest source of variation associated with broomrape tolerance and plant productivity. In contrast, PC2 explained a smaller but still substantial proportion of the variation (23.6%), reflecting differences in anatomical and physiological adaptation mechanisms among genotypes.

**Table 5:** Eigenvalues and percentage of variance explained by principal components.

PCA	Eigenvalue	Variance Explained (%)	Cumulative Variance (%)
PC1	23.77	69.90	69.90
PC2	8.02	23.60	93.50
PC3	1.21	3.56	97.06
PC4	0.54	1.59	98.65
PC5	0.28	0.82	99.47
PC6	0.13	0.38	99.85
PC7	0.04	0.12	99.97
PC8	0.01	0.03	100.00

**Fig. 4:**
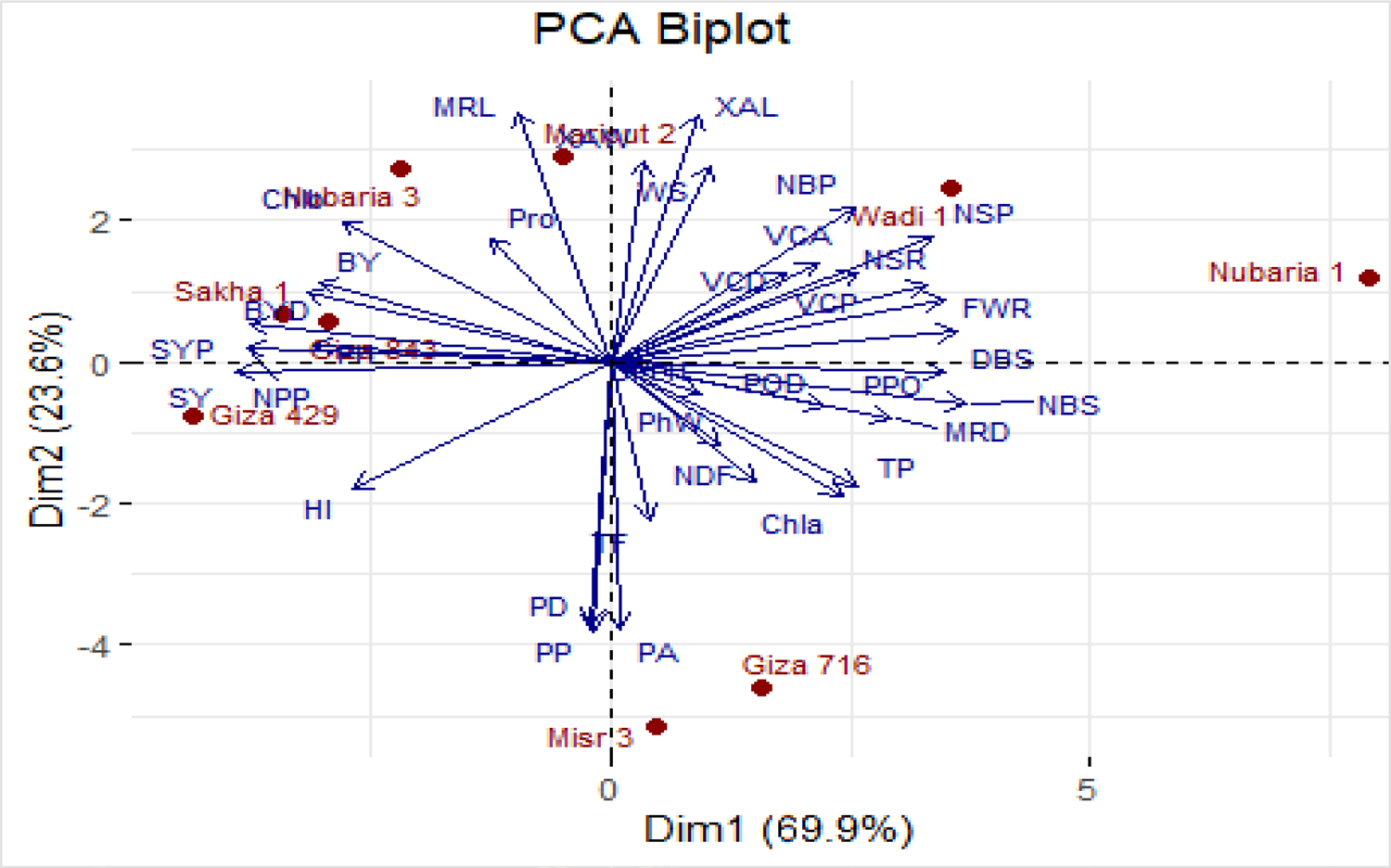
PCA biplot illustrating the multivariate relationships among Faba bean genotypes and measured agro-physiological, yield, and Bean Broomrape-related traits under infestation conditions. Red points represent Faba bean genotypes. PH = Plant Height; NB = Number of Branches per Plant; DF = Days to 50% Flowering; DM = Days to Maturity; FWR = Fresh Weight of Root; MRL = Main Root Length; MRD = Main Root Diameter; NSR = Number of Secondary Roots; WS = Weight of 100 Seeds; SY = Seed Yield per Plant; BY = Biological Yield; SYP = Seed Yield per Hectare; STY = Straw Yield; HI = Harvest Index; NBP = Number of Bean Broomrape Spikes; DWB = Dry Weight of Broomrape; TP = Total Phenolics; TF = Total Flavonoids; DPPH = 2,2-Diphenyl-1-picrylhydrazyl Radical Scavenging Activity; Pro = Proline Content; PPO = Polyphenol Oxidase Activity; POD = Peroxidase Activity; Chla = Chlorophyll a; Chlb = Chlorophyll b; PD = Pith Diameter; VCD = Vascular Cylinder Diameter; VCA = Vascular Cylinder Area; VCP = Vascular Cylinder Perimeter; XAL = Xylem Arm Length; XAW = Xylem Arm Width; PhW = Phloem Width; XVA = Xylem Vessel Area.

The PCA biplot (Fig. 4) revealed that most variables were represented by relatively long vectors, indicating strong contributions to genotype discrimination and high-quality representation within the PCA space ^35^. Traits associated with plant vigor and productivity, including SY, biological yield (BY), biomass production, plant height (PH), chlorophyll content, vascular tissue dimensions, antioxidant activity, and phenolic compounds, were positively associated with PC1. Conversely, harvest index (HI), pith diameter (PD), number of pods (PP), and number of pods per Plant (NPP) were negatively associated with this axis. The clustering of yield-related, anatomical, and biochemical traits on the positive side of PC1 suggests that these variables contribute to tolerance against broomrape infestation ^18^.

The strong association between yield traits and vascular characteristics supports the hypothesis that tolerant genotypes maintain superior resource acquisition and translocation despite parasite attack. Previous studies have shown that broomrape consumes water, minerals, and photoassimilates, reducing host growth and productivity. Therefore, genotypes with larger vascular cylinder diameters, wider xylem vessels, and thicker phloem tissues may support more efficient water and nutrient transport, thereby reducing the detrimental effects of parasitism. The positioning of resistant cultivars, particularly Giza 429 and Misr 3, near these traits in the PCA space further supports this interpretation. The positive association of total phenols, flavonoids, and antioxidant activity with PC1 suggests that biochemical defense mechanisms play a critical role in limiting broomrape damage. Phenolic compounds are known to strengthen cell walls through lignification and may act as physical or chemical barriers that restrict parasite penetration and development. Enhanced antioxidant activity may also alleviate oxidative stress induced by host–parasite interactions by scavenging ROS generated during infestation.

**Fig. 5:**
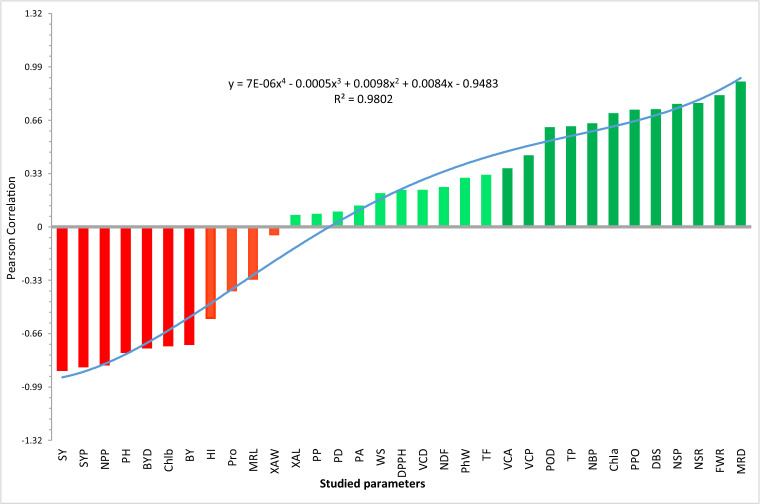
Correlation coefficients between the number of bean broomrape spikes (spikes/m^2^) and the other studied morphological, physiological, biochemical, and yield parameters of faba bean cultivated under naturally infested soil conditions. Positive correlations are represented by green bars, while negative correlations are represented by red bars, with the fitted polynomial trend line illustrating the overall association pattern among the evaluated traits. PH = Plant Height; NB = Number of Branches per Plant; DF = Days to 50% Flowering; DM = Days to Maturity; FWR = Fresh Weight of Root; MRL = Main Root Length; MRD = Main Root Diameter; NSR = Number of Secondary Roots; WS = Weight of 100 Seeds; SY = Seed Yield per Plant; BY = Biological Yield; SYP = Seed Yield per Hectare; STY = Straw Yield; HI = Harvest Index; NBP = Number of Bean Broomrape Spikes; DWB = Dry Weight of Broomrape; TP = Total Phenolics; TF = Total Flavonoids; DPPH = 2,2-Diphenyl-1-picrylhydrazyl Radical Scavenging Activity; Pro = Proline Content; PPO = Polyphenol Oxidase Activity; POD = Peroxidase Activity; Chla = Chlorophyll a; Chlb = Chlorophyll b; VCD = Vascular Cylinder Diameter; VCA = Vascular Cylinder Area; VCP = Vascular Cylinder Perimeter; XAL = Xylem Arm Length; XAW = Xylem Arm Width; PhW = Phloem Width; XVA = Xylem Vessel Area.

Pearson’s correlation analysis indicates that the number of bean broomrape spikes, which represents the severity of Orobanche crenata infestation, was strongly associated with several morphological, yield, anatomical, and biochemical traits (Fig. 5). NBS showed significant positive correlations with mean root diameter (MRD; r = 0.89), fresh root weight (FWR; r = 0.81), number of secondary roots (NSR; r = 0.76), and number of spikes per Plant (NSP; r = 0.76). These relationships indicate that increased parasite emergence was accompanied by enhanced parasite establishment and development. The positive association of NBS with root-related traits suggests that larger or more branched root systems may provide additional attachment sites for broomrape seeds, facilitating haustorial penetration and parasite growth^27,36^.

NBS exhibited strong negative correlations with seed yield (SY; r =  − 0.88), seed yield per plant (SYP; r =  − 0.86), number of Bean broomrape spikes (NBP; r =  − 0.85), plant height (PH; r =  − 0.77), biological yield (BY; r =  − 0.72), and chlorophyll b content (Chlb; r =  − 0.73). These findings demonstrate that increasing broomrape infestation reduced plant growth and productivity. The strong inverse relationship between NBS and yield-related traits confirms the detrimental impact of O. crenata on host performance, as the parasite acts as a strong sink for water, nutrients, and assimilates. Consequently, genotypes with lower NBS values maintained greater biomass production and reproductive capacity, highlighting their superior tolerance under infested conditions.

NBS was also positively correlated with several defense-related biochemical traits, including polyphenol oxidase activity (PPO; r = 0.72), peroxidase activity (POD; r = 0.61), total phenols (TP; r = 0.62), chlorophyll a (Chla; r = 0.70), and DPPH antioxidant activity (r = 0.22). These associations suggest that broomrape infestation induces oxidative stress and activates host defense mechanisms. Phenolic compounds and antioxidant enzymes play important roles in strengthening cell walls, lignification processes, and scavenging reactive oxygen species generated during host–parasite interactions. However, the positive correlations between NBS and these defense-related traits indicate that their accumulation is primarily a response to increasing infestation pressure rather than a complete barrier against parasite development. Therefore, biochemical defenses alone may not be sufficient to confer resistance unless combined with other tolerance mechanisms.

Regarding anatomical characteristics, NBS showed positive correlations with vascular cylinder diameter (VCD; r = 0.22), vascular cylinder area (VCA; r = 0.36), and vascular cylinder perimeter (VCP; r = 0.44). These relationships suggest that broomrape infestation is associated with modifications in host vascular tissues, likely reflecting adaptive responses aimed at maintaining water and nutrient transport despite the parasite’s resource withdrawal. Because O. crenata establishes direct vascular connections with its host, the development of larger vascular tissues may help sustain translocation processes under infestation stress. This interpretation is consistent with the PCA results, which identified vascular anatomical traits as major contributors to genotype tolerance^37^ see Fig. 5.

## Discussion

The proposed tolerance Faba bean hypothesis is based on several interconnected mechanisms, including: (i) reduced stimulation of broomrape germination through lower production of germination-inducing compounds in host root exudates; (ii) reduced lateral root formation, which minimize potential attachment sites for the parasite; (iii) delayed parasite attachment and emergence, thereby reducing the duration and severity of host-parasite interaction; and (iv) activation of structural defense responses such as lignification and vascular occlusion that restrict parasite penetration and vascular connection establishment. The diversity observed among the studied Faba bean genotypes in both shoot and root morphological and anatomical traits offers valuable clues as to why some varieties are more susceptible to broomrape than others^36^. Since broomrape is a root holoparasite that depends entirely on attaching to the host plant’s roots to survive and develop, characteristics such as root structure and the timing of root development play a crucial role in determining how vulnerable a plant might be^37^.

Noticeable differences in above-ground traits, such as plant height, branch number, and timing of developmental stages, highlight genetic diversity across cultivars and can indirectly affect how plants interact with the parasite^38^. For instance, Giza 429, which reached the tallest height (73 cm), and Nubaria 1, which produced the highest number of branches (4.66), likely had greater leaf area for photosynthesis. This increased energy production may support the Plant’s growth and potentially benefit the parasite once it is attached^39^. Wadi 1, which flowered in just 48.3 days and matured by 149.7 days, completed its life cycle faster than other cultivars. This shorter development period may help it sidestep the peak window when Bean Broomrape typically emerges and attaches, using a defense known as phenological escape, in which early flowering and maturation reduce the Plant’s exposure to the parasite^40^. Root traits, however, are more directly tied to how broomrape interacts with its host. Nubaria 1, for instance, produced the highest fresh root weight (39.3 g) and the greatest number of secondary roots (42.3), potentially offering a larger root surface area for broomrape seed germination and attachment^41^. Likewise, Nubaria 3, which developed the longest roots (16.83 cm), may have roots that reach deeper into, or more broomrape-infested, soil layers, increasing the risk of parasitic contact^42^.

**Fig. 6:**
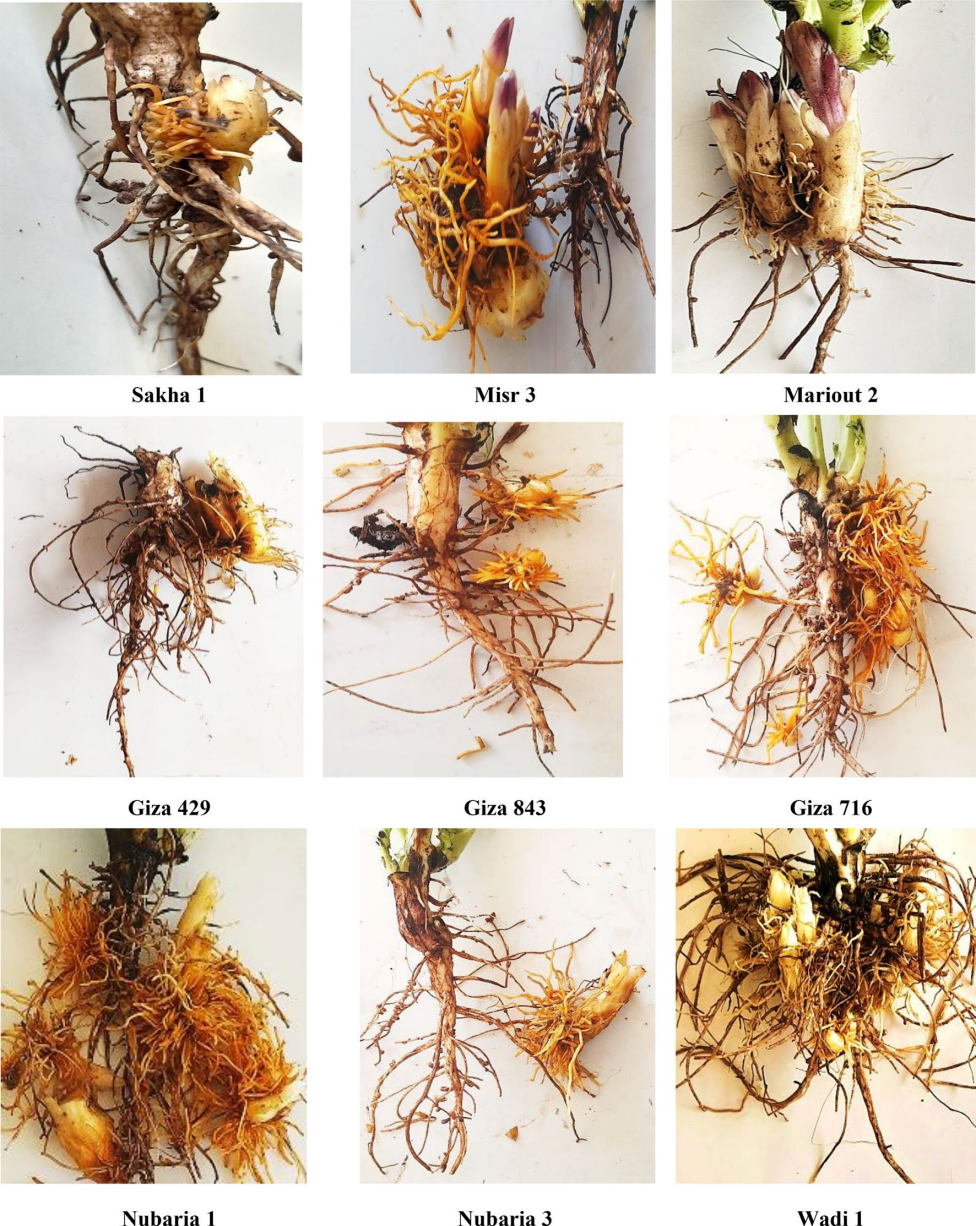
Root infection status of Faba bean (*V. faba* L.) genotypes in response to infestation by Bean Broomrape(*O. crenata*). The figure illustrates the degree of root attachment and infection observed in the evaluated genotypes, including Sakha 1, Misr 3, Mariout 2, Giza 429, Giza 843, Giza 716, Nubaria 1, Nubaria 3, and Wadi 1. Visual differences among genotypes reflect their relative susceptibility or tolerance to Bean Broomrape, as indicated by variation in the number, size, and development of Bean Broomrape tubercles attached to the host roots under the experimental conditions.

In contrast, Sakha 1 and Misr 3 had lower root mass and fewer lateral roots, which might make them less accessible to Bean Broomrape (Fig. 6), suggesting they could avoid infestation through root architectural traits. This reduced exposure could explain the lower parasite load in these cultivars^41^. Interestingly, Wadi 1 also had the thickest primary root diameter (1.9 cm), suggesting structural features that physically block or slow broomrape penetration. Thicker roots are often associated with greater suberization or lignification, natural defense processes that help plants resist parasite invasion. On the other hand, Sakha 1’s thinner, less-branched roots may limit its ability to meet both its own growth needs and those of an attached parasite. While this could help limit broomrape attachment, it might also compromise the Plant’s overall performance under infestation pressure^43^.

Giza 429 consistently delivered the highest performance, producing the highest seed yield per plant (20.88 g), the highest biological yield (10.40 t/ha), and the highest parasitized seed yield (3.18 t/ha). These results suggest that Giza 429 can maintain productivity even under heavy parasitic stress. Its strong pod and seed yield hint at possible structural or physiological resistance mechanisms that may prevent broomrape from fully establishing or lessen its impact once attached^44^. Nubaria 1 showed the lowest productivity, with seed yields dropping to 4.71 g per Plant and to just 0.87 t/ha under infestation. This cultivar also recorded the highest Bean Broomrape spike density and biomass, indicating a high level of susceptibility to the parasite. Differences in harvest index and straw yield across the cultivars reveal how each variety allocates its resources under stress. Tolerant types seemed to maintain a more balanced energy distribution between growth and reproduction^45^. Misr 3 and Nubaria 1 showed high TPC levels, indicating strong activation of the phenylpropanoid pathway, a key defense mechanism plants use to combat parasitic weeds^46^. These two genotypes also showed higher antioxidant capacity and lower proline^47^.

The role of enzymatic antioxidants, especially PPO and POD, provided further insight into the Plant’s defense systems^48^. Nubaria 1 had the highest activity levels of both enzymes, suggesting that its defense system includes robust oxidative responses that could help restrict Bean Broomrape through processes such as lignification and detoxification of harmful reactive oxygen species. Similar increases in peroxidase and antioxidant enzyme activities under stress conditions have been reported in legumes exposed to both biotic and abiotic stressors, in which enhanced POD and related antioxidant systems contributed to stress tolerance and cellular protection^48^. In addition, POD enzymes is known to participate in lignification, the oxidation of phenolic compounds, and the detoxification of reactive oxygen species generated during stress responses^49,50^. In contrast, Giza 843 showed the lowest enzymatic activity and the weakest antioxidant potential.

Chlorophyll content across genotypes showed less variation overall. Still, a noticeable drop in chlorophyll b in Nubaria 1 and Giza 716 suggested a compromise in photosynthetic efficiency, even in cultivars with strong enzymatic defense. Similar reductions in chlorophyll pigments under stress conditions have been documented in legumes, where oxidative stress negatively affected photosynthetic pigments despite the activation of antioxidant defense systems^51,52^. These findings underscore the value of using biochemical indicators, such as PPO and POD activity, alongside physiological markers to assess the tolerance of Faba bean cultivars to Bean Broomrape. This approach can help guide breeding and selection efforts in areas where the parasite is a persistent threat^53^. Cultivars such as Giza 719 and Giza 429 exhibited larger vascular bundle areas and perimeters, suggesting enhanced transport capacity and greater structural support. Larger vascular bundles are often associated with improved water and nutrient conduction and greater mechanical reinforcement, which can contribute to growth vigor and lodging resistance^54^. Similarly, xylem arm length and width varied significantly, with cultivars such as Giza 429 and Wadi 1 exhibiting longer and wider xylem arms. These features are critical for efficient water conduction. They may suggest adaptation to high transpiration rates or growth potential, as xylem dimensions have been shown to correlate with higher hydraulic conductivity and drought tolerance in many crop species^55^.

**Fig. 7:**
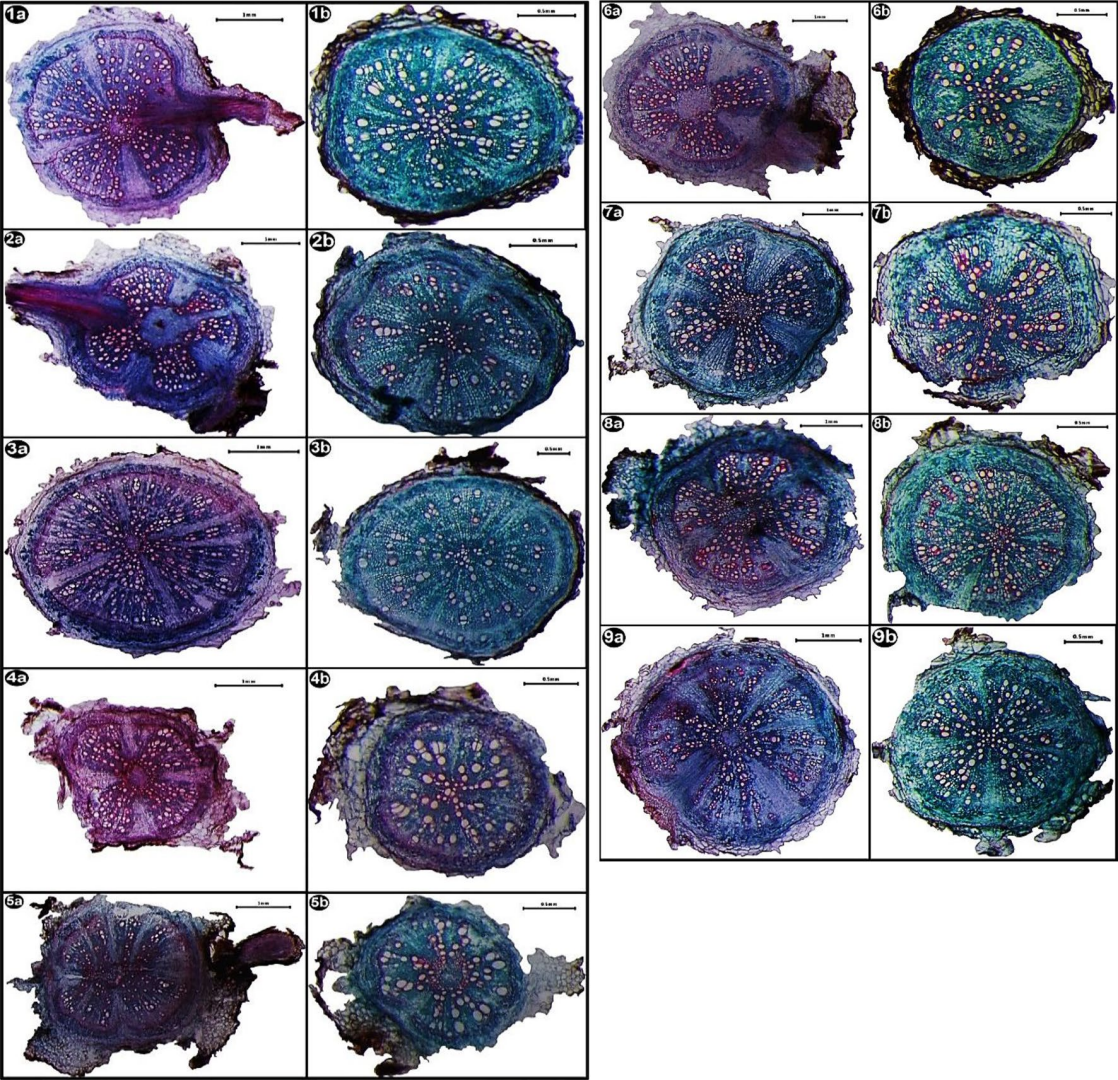
Photomicrographs of transverse root sections:(**a**) Main root;(** b**) Lateral root of the cultivated Faba bean genotypes under Bean Broomrape-infested soil conditions. Giza 843 (1), Nubaria 1 (2), Giza 429 (3), Giza 716 (4), Misr 3 (5), Sakha 1 (6), Nubaria 3 (7), Mariout 2 (8), Wadi 1 (9).

Wider phloem was detected in some cultivars, such as Misr 3 and Wadi 1, which may reflect increased assimilate transport capacity, vital for supporting vascular tissues during growth and development. Phloem anatomy influences plant responses to stress and injury^56^. Central root anatomy, chiefly pith characters, including pith diameter, area, and perimeter, showed presence-absence patterns across cultivars. Cultivars such as Giza 843 and Sakha 1 had large, well-developed piths, while others lacked pith. This variability suggests possible shifts in root structural significance, such as replacing storage parenchyma with supportive tissues, such as sclerenchyma, in narrower roots^57^. A more developed pith could enhance root elasticity and internal buffering capacity (Fig. 7). The Giza 429 cultivar displayed mostly large lateral root xylem vessels (7699 µm^2^), which could facilitate haustorial connections by the parasite, thereby enhancing susceptibility. This aligns with findings on vetch (*Vicia* spp.), where susceptible cultivars displayed larger xylem vessels, providing more accessible pathways for *Orobanche* penetration^58^.

On the contrary, cultivars displaying smaller xylem vessel areas may prevent parasite establishment. In the Faba bean (*V. faba*), resistance to *O. crenata* has been associated with anatomical structures, such as reduced vessel diameter and reinforced cell walls, that impede parasite entry. With lignification and callose deposition, these structural defenses block the growth of haustorial connections, conferring resistance^59^. Disease tolerance is a complex trait regulated by multiple anatomical, physiological, and biochemical parameters, even though larger xylem vessels are often associated with greater susceptibility, as they may facilitate faster pathogen movement through the vascular system. Despite having larger xylem vessels, Giza 429’s high tolerance may be attributed to enhanced defense mechanisms, including strengthened cell walls, increased phenolic compound accumulation, effective pathogen compartmentalization, and stronger induced defense responses. Similar results were reported by Pérez-de-Luque et al.^21^, who showed that resistance may be linked to cell wall reinforcement and structural changes that limit pathogen penetration and propagation, independent of vessel size. Xylem diameter cannot be regarded as a definitive indicator on its own. Consequently, the cultivars Giza 429 and Giza 843 exhibited lower broomrape spike density and reduced parasite dry matter accumulation compared with susceptible cultivars. This reduced parasitic pressure likely enabled these cultivars to maintain superior growth and productivity, resulting in higher biological, seed, and straw yields under broomrape-infested conditions^59^.

The proximity of these biochemical variables to the resistant genotypes in the PCA biplot and the correlation matrix reflects the role of antioxidant defense mechanisms in tolerance. Susceptible cultivars (Nubaria 1) were related to greater broomrape biomass and infestation characteristics, separating from yield, anatomical and biochemical defense factors^17^. This distinction indicates that susceptibility is characterized by reduced vascular development, poorer assimilate allocation and lower antioxidant defences, resulting in higher parasite establishment and yield losses. This hypothesis is supported by the noted reduction in seed yield obtained from Nubaria 1. The correlation analysis is consistent with the fundamental premise of this work that tolerance to O. crenata is a complex feature involving several interacting processes^10^. Genotypes with lower NBS values usually exhibited better growth and yield performance, as well as favorable anatomical and physiological traits. The data suggest that good tolerance requires the capacity to restrict parasite establishment, maintain photosynthetic activity and vascular function, and trigger biochemical defensive mechanisms^60^.

## Conclusion

This study offers valuable insights into the biological and root anatomical factors influencing Faba bean susceptibility or tolerance to broomrape (*O. crenata*) infestation. Giza 429 shows the highest tolerance level. It achieved strong yield performance (3.2 t/ha), experienced less Bean Broomrape attachment, and demonstrated a more active antioxidant defense system. Biochemical analysis showed that tolerant cultivars, such as Giza 429 and Misr 3, had higher phenolic levels, more potent antioxidant activity, and lower proline accumulation, indicating a more effective stress response. In addition, anatomical studies revealed that these tolerant genotypes had more developed root structures, including larger vascular cylinders, wider xylem vessels, and thicker phloem tissue. These features likely improve nutrient flow and create more substantial physical barriers to parasite entry. In the future, research should aim to identify genetic markers associated with resistance to support the development of more resilient Faba bean varieties.

## Data Availability

All obtained or analyzed data during this study are included in the manuscript.
